# Familial hypercholesterolaemia with early-onset coronary artery disease and recurrent in-stent restenosis associated with the LDLR gene c.428G>A mutation: a case report

**DOI:** 10.3389/fcvm.2025.1573543

**Published:** 2025-06-09

**Authors:** Chengpeng Zhang, Hui Li, Wei Zhang, Jian Huang, Jing Zhu

**Affiliations:** Department of Cardiology, The Second Affiliated Hospital of Soochow University, Suzhou, China

**Keywords:** familial hypercholesterolaemia, LDL receptor gene mutation, early-onset coronary artery disease, recurrent in-stent restenosis, PCSK9 inhibitors, precision medicine

## Abstract

**Background:**

Familial hypercholesterolaemia (FH) is characterised by significantly elevated low-density lipoprotein cholesterol (LDL-C) levels and early-onset coronary artery disease. Additionally, clopidogrel resistance is observed in approximately 30%–50% of individuals globally. Among FH patients with early-onset coronary artery disease, inadequate LDL-C management and suboptimal antiplatelet therapy after stent implantation are key factors contributing to recurrent in-stent restenosis (ISR).

**Case presentation:**

A 65-year-old male with a history of coronary artery disease (CAD), hyperlipidemia, and prior angioplasty presented to our institution with exacerbation of angina symptoms. The patient's CAD was initially diagnosed at age 52 (early-onset), with subsequent coronary angiography performed at Lianshui County Hospital. Coronary angiography confirmed coronary artery disease, prompting percutaneous coronary intervention (PCI) with stent placement: one in the right coronary artery and another in the left circumflex artery. Despite receiving standard antiplatelet (aspirin enteric-coated tablets 100 mg, clopidogrel 75 mg) and lipid-lowering therapy (pitavastatin calcium 2 mg), his LDL-C levels remained poorly controlled, and chest pain recurred. At the age of 62 and 65, he developed ISR with additional coronary artery lesions, necessitating balloon angioplasty. FH gene sequencing and clopidogrel resistance testing found he have a heterozygous LDL receptor (LDLR) gene mutation (c.428G>A, p.Cys143Tyr) and a clopidogrel genotype of CYP2C19 *1/*2. Based on these findings, his antiplatelet and lipid-lowering therapies were adjusted (aspirin 100 mg, clopidogrel 150 mg, rosuvastatin 10 mg, ezetimibe 10 mg and alirocumab 150 mg biweekly). Follow-up revealed that his LDL-C levels reached target values, and he remained asymptomatic. One year later, coronary angiography showed no disease progression, and the patient experienced no recurrence of chest pain. This case highlights the efficacy of precision treatment.

**Conclusions:**

For FH patients with early-onset CAD who are intolerant to ticagrelor, early implementation of FH genetic sequencing and clopidogrel genotyping is critical for personalised treatment.

## Introduction

Coronary artery disease (CAD) remains a leading cause of mortality globally. Familial hypercholesterolaemia (FH) is a hereditary disorder characterised by elevated cholesterol levels. It is classified into two forms: heterozygous FH (HeFH) and homozygous FH (HoFH). While HeFH represents the more common variant, HoFH is exceptionally rare and generally associated with unfavorable clinical outcomes. The present case involves a patient diagnosed with HeFH. Research revealed that HeFH increases the risk of CAD by 10–20 times compared to the general population (1). Furthermore, HeFH patients with pathogenic gene mutations have a threefold higher likelihood of developing early-onset CAD than those without such mutations (1). Advances in interventional techniques have made PCI and stent implantation standard treatments for CAD. However, in-stent restenosis (ISR) remains a significant limitation to the long-term success of PCI, particularly in patients with FH. Elevated low-density lipoprotein cholesterol (LDL-C) levels exacerbate vascular smooth muscle cell proliferation, increasing ISR risk. This report discusses a case of recurrent ISR in an FH patient with inadequate LDL-C control, examining the underlying mechanisms and potential therapeutic interventions. The objective is to present a previously unreported gene mutation: a G>A substitution at base 428 in exon 4, resulting in a p.Cys143Tyr amino acid substitution and to provide targeted guidance on the selection of lipid-lowering medicines and the application of antiplatelet therapy in HeFH patients with ISR.

## Case presentation

**Figure 1:**
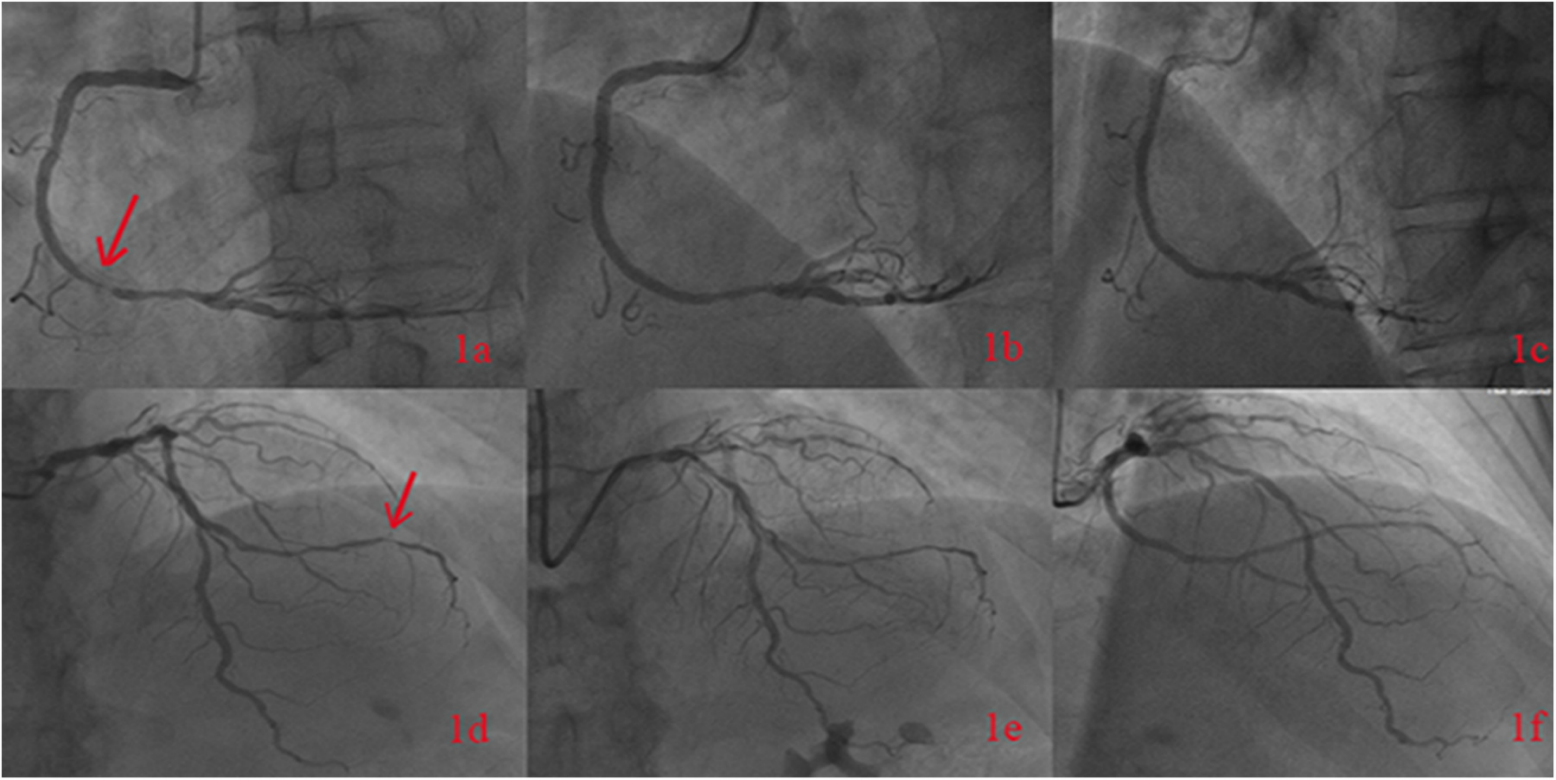
The figure shows the patient's coronary angiography in November 2023, indicating 99% stenosis in the distal segment of the right coronary artery with a stent **(a)**. Post-balloon angioplasty angiography of the right coronary artery was performed **(b)**, and follow-up angiography in October 2024 revealed no progression of RCA lesions **(c)**. The coronary angiography in November 2023 showed a 95% stenosis in the distal segment of the left circumflex artery **(d)**. Post-balloon angioplasty angiography was performed **(e)**, and follow-up angiography in October 2024 showed the LCX demonstrated mild in-stent neointimal hyperplasia without progression **(f)**.

**Figure 2:**
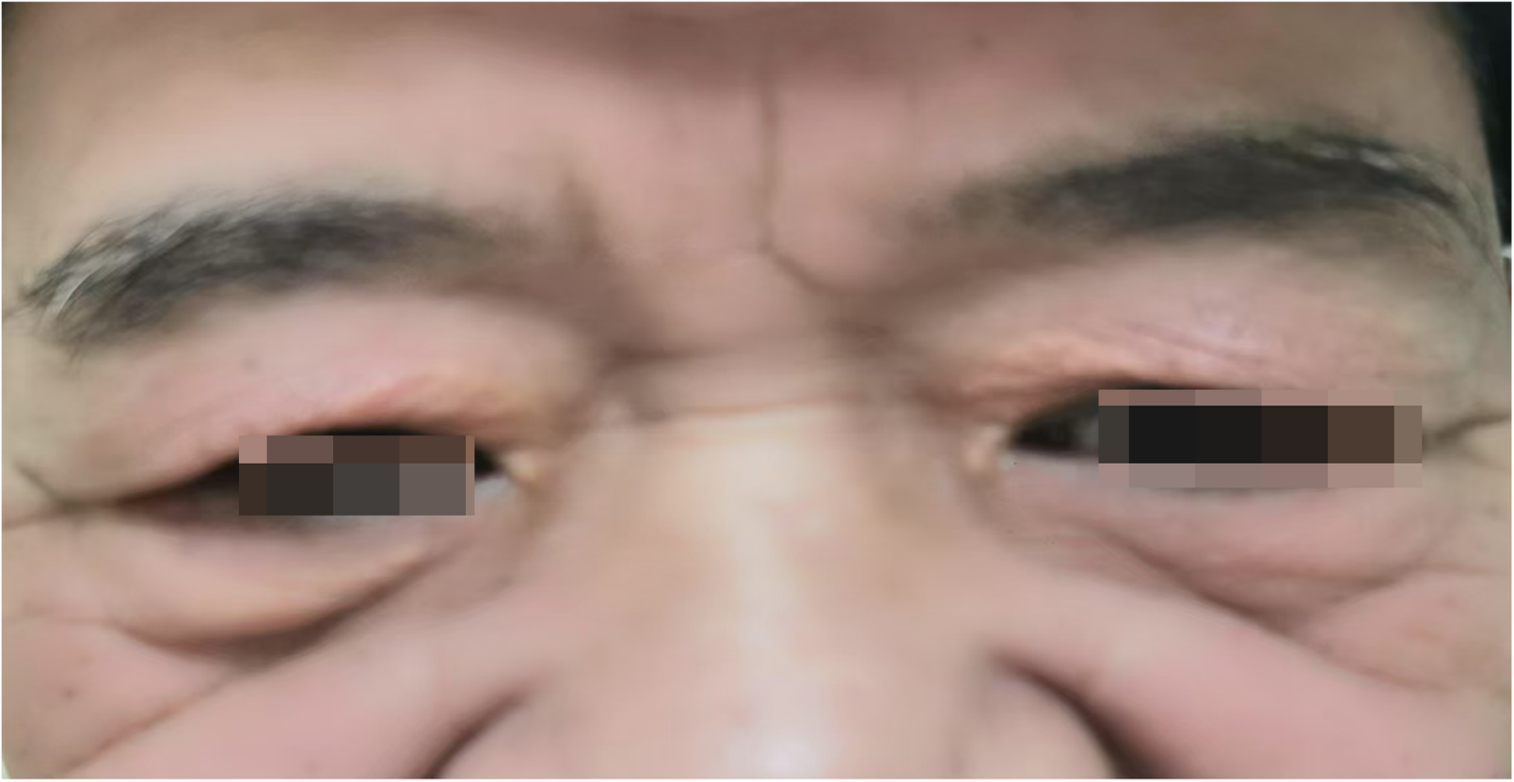
Xanthelasma.

**Figure 3:**
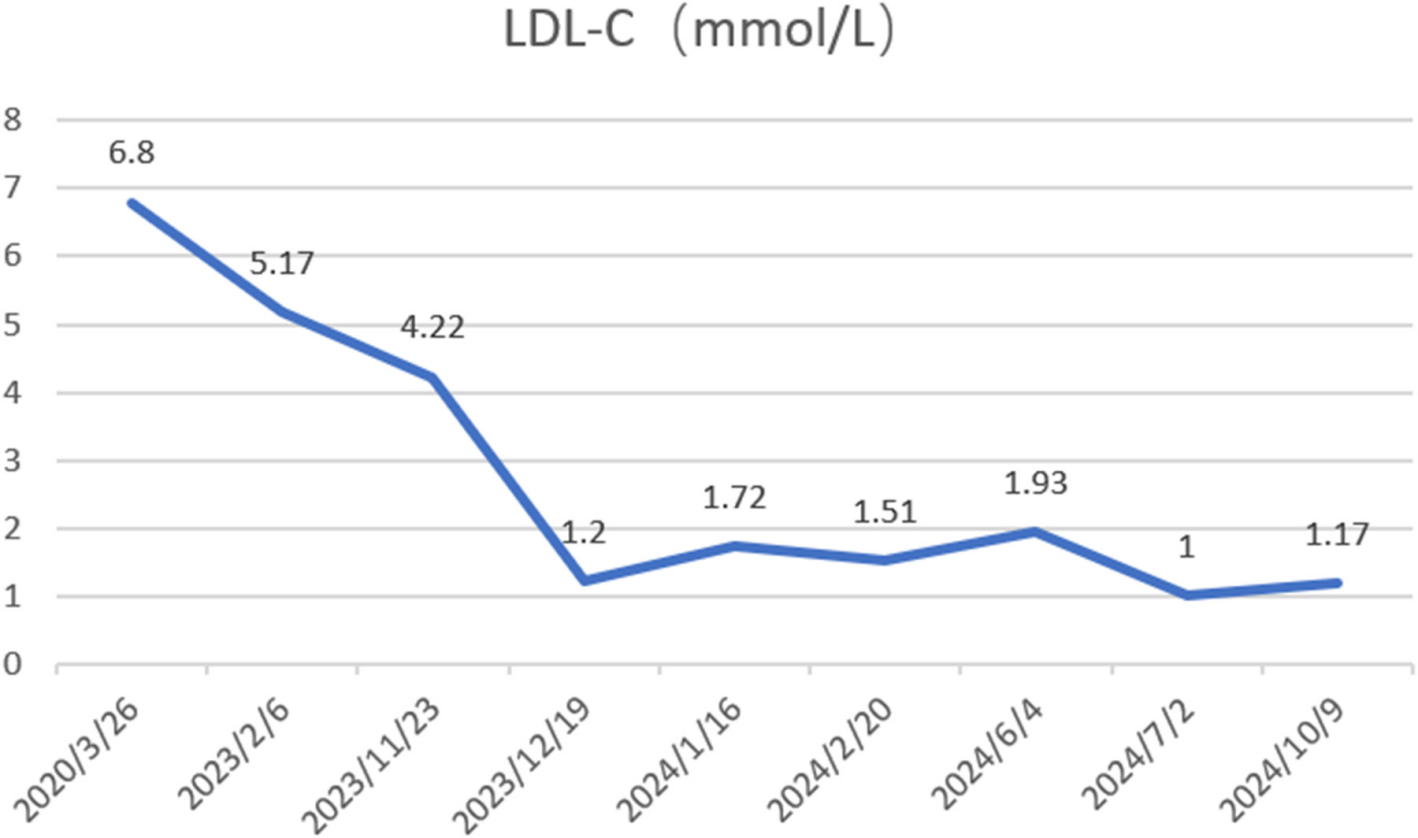
Changes in low-density lipoprotein cholesterol (LDL-C) levels were assessed before and after initiating alirocumab (150 mg every 2 weeks) on November 23, 2023.

A 65-year-old male was admitted to the Department of Cardiology at the Second Affiliated Hospital of Soochow University on 22 November 2023, with a 13-year history of chest tightness and pain, which had worsened in the past month. Thirteen years prior, he was diagnosed with early-onset CAD and underwent coronary angiography at Lianshui County People's Hospital. The procedure revealed significant stenosis requiring PCI with stent implantation in both the right coronary artery (RCA) and left circumflex artery (LCX). He was prescribed aspirin enteric-coated tablets (100 mg), clopidogrel (75 mg), and pitavastatin calcium (2 mg). Three years ago, the patient presented with chest pain at Zhongda Hospital Southeast University. Follow-up coronary angiography revealed plaque infiltration in the RCA, with a long proximal lesion (90% stenosis) and total occlusion in the mid-segment. Two additional drug-eluting stents were implanted in the RCA. Angiography also identified 30% stenosis in the distal left main artery, 70% stenosis with calcification in the proximal and mid-left anterior descending artery (LAD), and 60% stenosis in the mid-left circumflex artery (LCX), and 75% stenosis in the distal LCX. After discharge, the patient continued aspirin (100 mg), clopidogrel (75 mg), and pitavastatin calcium (2 mg). One month ago, the patient experienced recurrent chest pain. Coronary angiography showed diffuse in-stent proliferation in the proximal and mid-RCA and chronic total occlusion in the LCX stent. Balloon angioplasty was performed following recanalisation. Post-discharge, the patient's therapy was adjusted to aspirin (100 mg), clopidogrel (75 mg), rosuvastatin (10 mg), and ezetimibe (10 mg). However, LDL-C levels remained elevated, and chest pain recurred. Upon admission, the patient's symptoms worsened, including chest pain at rest and profuse sweating. The patient was diagnosed with unstable angina. Laboratory tests indicated total cholesterol of 5.72 mmol/L, triglycerides of 1.78 mmol/L, LDL-C of 4.22 mmol/L, and a clopidogrel genotype of CYP2C19 *1/*2. Electrocardiogram findings revealed T-wave inversion in leads I, avL, V5, and V6. Coronary angiography showed 99% stenosis in the distal RCA stent (Figure 1a), 40% stenosis in the distal left main coronary artery, a 50%–70% stenotic lesion in the proximal and mid-LAD, and 95% stenosis in the distal LCX stent (Figure 1d). Balloon angioplasty was performed on the RCA and distal LCX stents, achieving patent RCA stent with residual stenosis <10% in the distal LCX and thrombolysis in myocardial infarction grade 3 flow (Figures 1b,e). Following the procedure, the patient did not experience chest pain. The patient's therapy was adjusted to rosuvastatin (10 mg), ezetimibe (10 mg), and alirocumab (150 mg biweekly), alongside aspirin (100 mg) and clopidogrel (150 mg). The patient exhibited xanthelasma (Figure 2), refractory hyperlipidaemia, recurrent ISR, and a family history of hyperlipidaemia. Whole exome sequencing revealed a heterozygous LDL receptor (LDLR) gene mutation (c.428G>A, p.Cys143Tyr), consistent with FH. Clinical features and atherosclerotic CVD risk stratification placed the patient in the very high-risk category, warranting LDL-C reduction to <1.4 mmol/L, with a >50% baseline reduction per the European Society of Cardiology guidelines (2). Post-discharge, the patient experienced no chest pain, and LDL-C levels remained at target (Figure 3). One year later, follow-up coronary angiography revealed no progression of RCA lesions (Figure 1c). The left main artery showed approximately 40% stenosis, and LAD stenosis persisted at 50%–70%. The LCX demonstrated mild in-stent neointimal hyperplasia without progression (Figure 1f). The patient remained asymptomatic, and no further ISR occurred. The patient's clinical course is shown in the table below (Table 1).

**Table 1:** The table above summarizes the patient's clinical course from 2010 to present.

The patient's medical visit history										
Date	Age	Hospital	Coronary angiography						Interventions	Lipid-lowering drugs
			LM	RCA			LAD	LCX		
				Proximal segment	Mid segment	Distal segment				
2010	52	Lianshui County People's Hospital	Unknow	Unknow	Unknow	Unknow	Unknow	Unknow	One stent was placesd in RCA,one stent in distal LCX, the type of stents are unknown	Pitavastatin 2 mg
2020	62	Zhongda Hospital Southeast University	30%	90%	Total occlusion	–	70%	75%	Two drug-eluting stents were placed in RCA	Rosuvastatin 10 mg
2023	65	The Second Affiliated Hospital of Soochow University	40%	Without progression	Without progression	99%	50–70%	95%	Balloon angioplasty was performed on the RCA and distal LCX stents	Rosuvastatin 10 mg, ezetimibe 10 mg, and alirocumab 150 mg biweekly
2024	66	The Second Affiliated Hospital of Soochow University	40%	Without progression	Without progression	Without progression	Without progression	Without progression	No interventions	Rosuvastatin 10 mg, ezetimibe 10 mg, and alirocumab 150 mg biweekly

## Discussion

FH is an autosomal dominant genetic disorder characterised by significantly elevated LDL-C levels, often accompanied by Achilles tendon xanthomas. In recent years, the incidence of premature myocardial infarction (PMI) has risen, with FH recognised as one of the most prevalent genetic disorders of cholesterol metabolism contributing to PMI. FH-related gene mutations are identified in approximately 7.6% of Chinese patients with PMI (3). Studies consistently demonstrate that most patients with FH fail to achieve the recommended LDL-C targets, resulting in a 13-fold increased risk of CAD (4).

**Figure 4:**
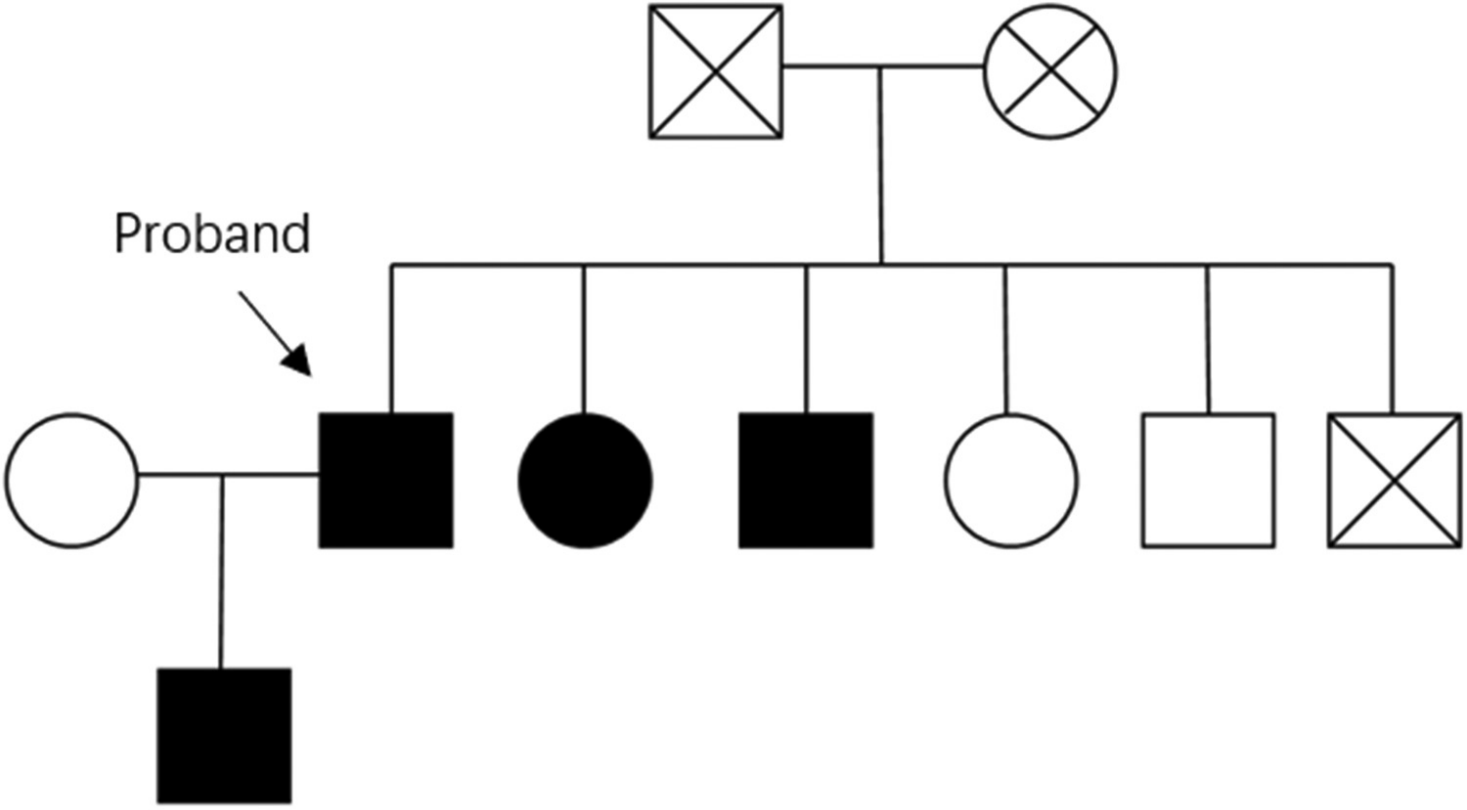
The pedigree illustrates the patient's family history, with fully shaded symbols denoting hyperlipidemia. No cases of coronary artery disease (CAD) were reported among the relatives.

The patient first developed CAD at 52 years of age, and inadequate lipid management prevented his LDL-C from reaching target levels. Additionally, the absence of clopidogrel genetic testing led to suboptimal antiplatelet therapy after PCI, resulting in recurrent coronary events at 62 and 65 years of age. Poor LDL-C control and inappropriate antiplatelet therapy were likely critical contributors to the recurrent coronary artery stenosis. Despite treatment with rosuvastatin (10 mg) and ezetimibe (10 mg), the patient's LDL-C levels remained above target for an extended period. Studies have shown that LDLR mutations in patients with heterozygous FH might reduce the efficacy of -hydroxy-3-methylglutaryl-coenzyme A reductase inhibitors (5). Genetic testing revealed a heterozygous LDLR gene mutation (c.428G>A), leading to a p.Cys143Tyr amino acid substitution. According to the American College of Medical Genetics and Genomics guidelines, this variant was initially classified as pathogenic. Unfortunately, the patient's parents, siblings, and offspring did not undergo FH genetic testing, leaving the origin of the pathogenic variant unknown. However, a family history of hyperlipidaemia and xanthomas was documented (Figure 4).

We identified two previously reported familial hypercholesterolemia (FH) cases from China sharing the same LDLR mutation as our patient: a father-daughter pair. The daughter exhibited compound heterozygosity for two missense mutations: c.428G>A (p.Cys122Tyr) in exon 4 and c.1211C>T (p.Thr383Ile) in exon 9. Her clinical presentation was severe, with markedly elevated LDL-C (13.3 mmol/L), cutaneous xanthomas, and electrocardiographic evidence of ischemia. The father, carrying only the c.428G>A (p.Cys122Tyr) variant, demonstrated a milder phenotype with LDL-C of 6.78 mmol/L and absence of xanthomas or ischemic changes. Neither patient underwent coronary angiography, precluding assessment of coronary artery disease severity. Furthermore, the absence of follow-up data limited evaluation of treatment efficacy and long-term outcomes (6).

A United States survey revealed that over 80% of patients with FH receiving maximal lipid-lowering therapy still failed to achieve LDL-C targets (7). This is because patients with familial hypercholesterolemia (FH) often exhibit limited responsiveness to statin therapy, and the degree of response can vary significantly depending on the specific genotype. A study from Canada demonstrated that the LDL-C-lowering effect of statins was significantly weaker in individuals with receptor-negative (RN) mutations compared to those with receptor-defective (RD) mutations. RN mutations include null alleles and transport-defective alleles, whereas RD mutations encompass binding-defective, internalisation-defective, and recycling-defective alleles. The study also highlighted the concept of residual LDL receptor (LDLR) activity and suggested that the effectiveness of statins in heterozygous FH (HeFH) patients appears to be positively correlated with the residual activity of the LDLR (8). As previously discussed, FH patients with different LDLR monogenic mutation genotypes exhibit varied responses to statin treatment. Clinically, some FH patients carry double heterozygous mutations. In a Warsaw study, patients with LDLR, APOB, or LDLR+APOB double heterozygous mutations received 12 weeks of alirocumab therapy. Monogenic FH patients demonstrated favorable responses to alirocumab, and their LDL-C reduction levels were comparable (about 60% reduction in LDL cholesterol levels). However, in patients with double heterozygous for mutations in LDLR and APOB genes, there was a hyporesponsiveness to alirocumab, and the reduction in LDL-C was only by 23%. Therefore, genetic diagnosis is crucial for optimizing lipid-lowering strategies and personalizing treatment in FH patients (9).

For patients with FH whose LDL-C remains uncontrolled after PCI, adding proprotein convertase subtilisin/kexin type 9 (PCSK9) inhibitors is essential. PCSK9 monoclonal antibodies effectively reduce LDL-C levels in patients with heterozygous and homozygous FH, enabling better achievement of treatment targets (10). The patient was started on alirocumab (150 mg every 2 weeks) with long-term monitoring of lipid levels. After 1 year, the patient's LDL-C levels reached treatment targets, achieving a reduction of >50%. However, it is notable that some patients with LDLR mutations exhibit a limited response to PCSK9 inhibitors (PCSK9is), as demonstrated in a case of a young female with an LDLR frameshift mutation who, despite treatment with multiple lipid-lowering medicines, including PCSK9is, experienced PMI (11). This young female have two mutation sites in the proband's LDLR gene and she was diagnosed with compound heterozygous FH. This may suggest that FH patients with compound heterozygosity show limited response to PCSK9 inhibitors. The efficacy of PCSK9is depends on LDLR activity, and loss-of-function mutations might result in poor therapeutic outcomes. Treatment plans for such patients should include early FH genetic testing, close monitoring of LDL-C levels, and timely adjustments to lipid-lowering strategies (11).

The patient could not tolerate ticagrelor due to respiratory distress, prompting an adjustment to aspirin (100 mg) and clopidogrel (75 mg). Genetic testing identified the patient's CYP2C19 genotype as *1/*2, indicating a moderate metaboliser status. This genotype is associated with reduced clopidogrel efficacy, and the standard 75 mg dose might not achieve optimal therapeutic effects, contributing to recurrent ISR. A study published in the Journal of the American Medical Association reported that carriers of the CYP2C19 loss-of-function allele face higher risks of cardiovascular death, myocardial infarction, and stroke, particularly for in-stent thrombosis events (12). These findings underscore the role of CYP2C19 polymorphisms in ISR recurrence. Additionally, a study in the Chinese population demonstrated that individualised antiplatelet therapy guided by CYP2C19 genetic testing significantly reduced the incidence of major adverse cardiovascular events (13). Following clopidogrel genetic testing, the antiplatelet regimen was adjusted to aspirin (100 mg) and clopidogrel (150 mg). Following adjustments to the lipid-lowering and antiplatelet therapies, the patient was followed up for 1 year. During this period, he remained asymptomatic, and his LDL-C levels were consistently within the target range. A follow-up coronary angiogram in October 2024 showed no progression of coronary stenosis, confirming the effectiveness of precision treatment.

## Conclusion

FH is strongly associated with early-onset CAD; however, many patients remain undiagnosed until recurrent severe cardiovascular events occur. Early FH genetic screening is crucial for patients with a family history of hyperlipidaemia and PMI to facilitate early detection and reduce the incidence of ISR. Unfortunately, the complexity of FH-related gene mutations limits current research from providing clinicians with genotype-specific guidance for selecting optimal medications in personalised treatment. Regular LDL-C monitoring and timely medication adjustments remain essential for effective disease management. Moreover, the differential efficacy of antiplatelet medicines in FH patients with various genotypes requires further investigation. For FH patients with early-onset CAD who are intolerant to ticagrelor, early implementation of FH genetic sequencing and clopidogrel genotyping is critical for personalised treatment. For patients whose LDL-C levels remain uncontrolled despite maximum lipid-lowering therapy, early initiation of PCSK9is is essential to achieve treatment targets, prevent recurrent ISR, and reduce CAD recurrence.

## Data Availability

The datasets presented in this study can be found in online repositories. The names of the repository/repositories and accession number(s) can be found in the article/Supplementary Material.

## References

[1] Séguro F Rabès JP Taraszkiewicz D Ruidavets JB Bongard V Ferrières J. Genetic diagnosis of familial hypercholesterolemia is associated with a premature and high coronary heart disease risk. Clin Cardiol. (2018) 41(3):385–91. 10.1002/clc.22881 29574850 PMC6489920

[2] Mach F Baigent C Catapano AL Koskinas KC Casula M Badimon L 2019 ESC/EAS guidelines for the management of dyslipidaemias: lipid modification to reduce cardiovascular risk. Eur Heart J. (2020) 41(1):111–88. 10.1093/eurheartj/ehz455. Erratum in: *Eur Heart J*. (2020) 41(44):4255. doi: 10.1093/eurheartj/ehz826 31504418

[3] Lee C Cui Y Song J Li S Zhang F Wu M Effects of familial hypercholesterolemia-associated genes on the phenotype of premature myocardial infarction. Lipids Health Dis. (2019) 18(1):95. 10.1186/s12944-019-1042-3 30971288 PMC6458678

[4] Nordestgaard BG Chapman MJ Humphries SE Ginsberg HN Masana L Descamps OS Familial hypercholesterolaemia is underdiagnosed and undertreated in the general population: guidance for clinicians to prevent coronary heart disease. Eur Heart J. (2013) 34(45):3478–90. 10.1093/eurheartj/eht273 23956253 PMC3844152

[5] Couture P Brun LD Szots F Lelièvre M Gaudet D Després JP Association of specific LDL receptor gene mutations with differential plasma lipoprotein response to simvastatin in young French Canadians with heterozygous familial hypercholesterolemia. Arterioscler Thromb Vasc Biol. (1998) 18(6):1007–12. 10.1161/01.atv.18.6.1007 9633944

[6] Wang L Lin J Liu S Cao S Liu J Yong Q Mutations in the LDL receptor gene in four Chinese homozygous familial hypercholesterolemia phenotype patients. Nutr Metab Cardiovasc Dis. (2009) 19(6):391–400. 10.1016/j.numecd.2008.07.011 19073363

[7] Pang J Chan DC Watts GF. The knowns and unknowns of contemporary statin therapy for familial hypercholesterolemia. Curr Atheroscler Rep. (2020) 22(11):64. 10.1007/s11883-020-00884-2 32870376 PMC7459268

[8] Roy G Couture P Genest J Ruel I Baass A Bergeron J Influence of the LDL-receptor genotype on statin response in heterozygous familial hypercholesterolemia: insights from the Canadian FH registry. Can J Cardiol. (2022) 38(3):311–9. 10.1016/j.cjca.2021.10.013 34774719

[9] Rogozik J Rokicki JK Grabowski M Główczyńska R. Gene mutation in patients with familial hypercholesterolemia and response to alirocumab treatment-a single-centre analysis. J Clin Med. (2024) 13(18):5615. 10.3390/jcm13185615 39337102 PMC11433266

[10] Ellis KL Pang J Schultz CJ Watts GF. New data on familial hypercholesterolaemia and acute coronary syndromes: the promise of PCSK9 monoclonal antibodies in the light of recent clinical trials. Eur J Prev Cardiol. (2017) 24(11):1200–5. 10.1177/2047487317708890 28482694

[11] Zhang Z Yang R Zhu J Yang X Luo H Wang H Failure of lipid control by PCSK9 inhibitors in compound heterozygous familial hypercholesterolemia complicated with premature myocardial infarction: a case report. Clin Case Rep. (2024) 12(3):e8498. 10.1002/ccr3.8498 38487640 PMC10939999

[12] Mega JL. Reduced-function CYP2C19 genotype and risk of adverse clinical outcomes among patients treated with clopidogrel predominantly for PCI: a meta-analysis. JAMA. (2010) 304(16):1821–30. 10.1001/jama.2010.1543 20978260 PMC3048820

[13] Shen DL Wang B Bai J Han Q Liu C Huang XH Clinical value of CYP2C19 genetic testing for guiding the antiplatelet therapy in a Chinese population. J Cardiovasc Pharmacol. (2016) 67(3):232–6. 10.1097/FJC.0000000000000337 26727381

